# COMPare: a prospective cohort study correcting and monitoring 58 misreported trials in real time

**DOI:** 10.1186/s13063-019-3173-2

**Published:** 2019-02-14

**Authors:** Ben Goldacre, Henry Drysdale, Aaron Dale, Ioan Milosevic, Eirion Slade, Philip Hartley, Cicely Marston, Anna Powell-Smith, Carl Heneghan, Kamal R. Mahtani

**Affiliations:** 1 Centre for Evidence-Based Medicine, Department of Primary Care Health Sciences, University of Oxford, Radcliffe Observatory Quarter, Woodstock Road, Oxford, OX2 6GG UK; 2 Department of Social and Environmental Health Research, London School of Hygiene and Tropical Medicine , Keppel St, Bloomsbury, London, WC1E 7HT UK

**Keywords:** Outcomes, Misreporting, Trials, CONSORT, Audit, ICMJE, Editorial conduct

## Abstract

**Background:**

Discrepancies between pre-specified and reported outcomes are an important source of bias in trials. Despite legislation, guidelines and public commitments on correct reporting from journals, outcome misreporting continues to be prevalent. We aimed to document the extent of misreporting, establish whether it was possible to publish correction letters on all misreported trials as they were published, and monitor responses from editors and trialists to understand why outcome misreporting persists despite public commitments to address it.

**Methods:**

We identified five high-impact journals endorsing Consolidated Standards of Reporting Trials (CONSORT) (*New England Journal of Medicine*, *The Lancet*, *Journal of the American Medical Association*, *British Medical Journal*, and *Annals of Internal Medicine*) and assessed all trials over a six-week period to identify every correctly and incorrectly reported outcome, comparing published reports against published protocols or registry entries, using CONSORT as the gold standard. A correction letter describing all discrepancies was submitted to the journal for all misreported trials, and detailed coding sheets were shared publicly. The proportion of letters published and delay to publication were assessed over 12 months of follow-up. Correspondence received from journals and authors was documented and themes were extracted.

**Results:**

Sixty-seven trials were assessed in total. Outcome reporting was poor overall and there was wide variation between journals on pre-specified primary outcomes (mean 76% correctly reported, journal range 25–96%), secondary outcomes (mean 55%, range 31–72%), and number of undeclared additional outcomes per trial (mean 5.4, range 2.9–8.3). Fifty-eight trials had discrepancies requiring a correction letter (87%, journal range 67–100%). Twenty-three letters were published (40%) with extensive variation between journals (range 0–100%). Where letters were published, there were delays (median 99 days, range 0–257 days). Twenty-nine studies had a pre-trial protocol publicly available (43%, range 0–86%). Qualitative analysis demonstrated extensive misunderstandings among journal editors about correct outcome reporting and CONSORT. Some journals did not engage positively when provided correspondence that identified misreporting; we identified possible breaches of ethics and publishing guidelines.

**Conclusions:**

All five journals were listed as endorsing CONSORT, but all exhibited extensive breaches of this guidance, and most rejected correction letters documenting shortcomings. Readers are likely to be misled by this discrepancy. We discuss the advantages of prospective methodology research sharing all data openly and pro-actively in real time as feedback on critiqued studies. This is the first empirical study of major academic journals’ willingness to publish a cohort of comparable and objective correction letters on misreported high-impact studies. Suggested improvements include changes to correspondence processes at journals, alternatives for indexed post-publication peer review, changes to CONSORT’s mechanisms for enforcement, and novel strategies for research on methods and reporting.

**Electronic supplementary material:**

The online version of this article (10.1186/s13063-019-3173-2) contains supplementary material, which is available to authorized users.

## Background

Discrepancies between pre-specified and reported outcomes are an important and widespread source of bias in clinical trials [1]. Where outcome misreporting is permitted, it increases the likelihood that reported differences have arisen through chance or are exaggerated [2, 3]. Clinical trial registers were established to address selective reporting [4] and require that all pre-specified outcomes are entered at the outset of the trial in a time-stamped and publicly accessible location. Registering clinical trials and pre-specifying their outcomes are now mandated by legislation in numerous territories, including the US [5], with strong support from global organisations, including the World Health Organization (WHO) [6], the International Committee of Medical Journal Editors (ICMJE) [7] and an extensive range of professional bodies, funders, ethics committees, publishers, universities and legislatures [8]. The importance of reporting all pre-specified outcomes and documenting changes is also emphasised in the International Conference on Harmonisation of Good Clinical Practice (ICH-GCP) [9] and the detailed Consolidated Standards of Reporting Trials (CONSORT) guidelines on best practice in trial reporting [10], which are endorsed by 585 academic journals [11].

However, despite near universal recognition of the importance of this issue and extensive public commitments to address the problem, trial reports in academic journals routinely fail to report pre-specified outcomes, and add in non-pre-specified outcomes, without disclosing that this has occurred. A 2015 systematic review [1] found 27 studies comparing pre-specified outcomes against those reported, in cohorts of between 1 and 198 trials (median *n* = 65 trials). The median proportion of trials with a discrepancy on primary outcomes was 31% (interquartile range (IQR) 17–45%). Eight studies also assessed the impact of outcome switching on the statistical significance of the published outcome and found that outcome switching favoured the reporting of significant outcomes in half the trials. However, owing to lack of access to all measured outcomes, this biased reporting could not be assessed in many cases and therefore the reviewers concluded that this figure was likely to be an underestimate. The most common issues identified were failure to report a pre-specified outcome, publication of a non-pre-specified outcome, reporting a pre-specified primary outcome as a secondary outcome, and a change in the timing of a pre-specified outcome.

In the Centre for Evidence-Based Medicine Outcome Monitoring Project (COMPare), we aimed to assess the prevalence of outcome misreporting, as in previous research, and to explore whether it was possible to publish correction letters on all trials with misreported outcomes in real time, as they were published, in order to ensure that the academic record was more CONSORT-compliant, as per journals’ public commitments. We also aimed to monitor responses from editors and trialists to this standardised set of correction letters, to better understand why outcome misreporting persists despite public commitments to address it, and to test the ability of academic journals to self-correct when breaches of their public commitments are reported.

## Methods

We set out to prospectively identify all trials published in five leading medical journals over a six-week period, identify every correctly and incorrectly reported outcome in every trial by comparing the published report against the published pre-trial protocol (or, where this was unavailable, the pre-trial registry entry), write a correction letter to the journal for publication on all misreported trials, and document the responses from journals. We used mixed methods combining quantitative analyses of the prevalence of flaws identified and quantitative and qualitative description of the responses of the journals to correspondence that notified them of misreporting. We used similar methods to assess the responses from trialists on the papers being assessed: these findings are reported in an accompanying paper.

### Sample

We prospectively selected five leading academic journals regularly publishing randomised controlled trials (RCTs) from those currently listed as endorsing the CONSORT guidelines: *New England Journal of Medicine* (NEJM), *The Lancet*, *Annals of Internal Medicine*, *Journal of the American Medical Association* (JAMA), and the *British Medical Journal* (BMJ). All trials published between 19 October and 30 November 2015 were included. This sample frame was selected as it was likely to yield a sample of trials comparable to the median sample size of all studies in the most current systematic review on outcome misreporting [1] and reflected what was practically achievable with our team size and availability.

### Coding of outcome reporting

Each published trial was allocated to one researcher (HD, AD, IM, ES and PH) who collected all relevant documents pertaining to that trial and archived them into a shared folder. This included the trial report, any appendices, a copy of the registry entry, and the trial protocol. As in previous work on outcome misreporting [1] and consistent with CONSORT’s requirements to declare all changes made after the start of a trial, we set out to identify the outcomes pre-specified before trial commencement. We searched initially for a published protocol dated before trial commencement or a subsequent protocol with a change log that allowed inference of pre-commencement outcomes. If this was not available, then we searched for a registry entry dated before trial commencement. If there were amendments to the registry entry, we accessed historical contents of the registry (for example, using the “archive” function on ClinicalTrials.gov) to find the most recent set of pre-specified outcomes dated before trial commencement. For each trial, the initial reviewer entered all pre-specified outcomes onto our data sheet for that study.

The trial report and appendices were then read in full and searched by the reviewer to establish whether each pre-specified outcome was reported and whether primary outcomes were reported as secondary outcomes (or vice versa) and to identify any novel non-pre-specified outcomes that were reported but not flagged as novel. The data sheet was updated accordingly. A single researcher reviewed and extracted data from each included trial, which then was checked and verified by a second. This data set (including the data sheet and the underlying documents) then was presented to one of the senior supervising clinical academics on the team (BG, CH and KM) where the data extraction was replicated in full. During this meeting (meetings typically lasted two or more hours and there were multiple meetings each week), all source documents and extracted pre-specified outcomes were identified and checked, and the location where each outcome was reported was identified in the paper. If outcomes were not reported and had not already been found by two researchers, then, at a minimum, key search terms were used as a check on the trial report and appendices, and all results tables were reviewed. Any discrepancies were resolved through discussion or, where needed, through referral to one of the other senior supervising clinical academics until consensus was reached. For each trial where outcome switching had occurred, the text of the correction letter to the journal was finalised in the team meeting, formally signed off by the supervising clinical academic, and submitted to the journal by the first reviewer before the submission deadline. Task allocation was closely managed by one team member (HD) as turnaround time from publication to submission was very short for some journals (for example, two weeks for *The Lancet* and three weeks for NEJM) and many trials were being assessed and responded to simultaneously.

CONSORT guidance states that trial publications should report “completely defined pre-specified primary and secondary outcome measures, including how and when they were assessed” (6a) and “any changes to trial outcomes after the trial commenced, with reasons” (6b) with further elaboration on these issues in the accompanying CONSORT publication [10]. Therefore, consistent with CONSORT, where outcome switching occurred but was openly declared to have occurred in the trial report, these outcomes were classified as correctly reported, as there are often valid reasons for reported outcomes to differ from those pre-specified.

### Letter preparation and submission

We constructed a set of template letters to match the journal word limits so that all letters were standardised and comparable (Additional file 1a–c). All journals’ instructions to authors were checked for the time limit and word limit on letters for publication to ensure that there were no grounds for rejection on these procedural issues. Letters reported only the fact of the outcome misreporting, and the breach of CONSORT guidelines, rather than any arguable issue of opinion that might differ between letters or otherwise impact on acceptance and responses. No comments were made on the authors’ background or possible motives for misreporting. We did not adjudicate on the validity of the reasons given for changing pre-specified outcomes. We did not give any subjective opinion on whether the outcome misreporting would lead to clinical harm and reported only the matter of fact: that the journal, having endorsed CONSORT, had breached the CONSORT trial reporting guidance.

All correspondence with journals was collected in a team email account and archived. Where all letters were rejected, this was contested. Where letters were published alongside trialists’ replies and these replies raised new issues or misunderstandings, we replied setting out our concerns. We aimed to conduct all correspondence extremely politely and respond on matters of fact. All outgoing correspondence was reviewed and co-authored by at least one supervising clinical academic and, in many cases, all three. At the conclusion of the study, we extracted themes and key issues in all correspondence in collaboration with a qualitative researcher (CM).

We created a bespoke website at COMPare-trials.org to archive all data and correspondence in public, reflecting a broader commitment to open science [12]. All underlying raw data sheets were shared in full as studies were added to the site. This allowed any interested party, including trialists and editors, to openly review or contest our coding of every outcome in every trial. An automatically updated table of findings calculated rolling summary statistics from the underlying raw data. Correspondence with journals was archived publicly on the site, including the initial letter submitted for publication (after a 4-week delay, to avoid letters’ being rejected on grounds of prior publication), alongside key incoming and outgoing correspondence with journals and trialists.

### Data analysis

We generated summary statistics, with confidence intervals and ranges as appropriate, on all outcomes. The follow-up period was 12 months from submission of the final letter. Our pre-specified primary outcomes were proportion of pre-specified outcomes reported, proportion of reported outcomes that are non-pre-specified and not declared as such, proportion of letters published in print, and publication delay in days. Our secondary outcomes were all of the primary outcomes, broken down by journal. At the end of the study, we added two outcomes that were not pre-specified in the original protocol. These were the number and proportion of trials with any discrepancy on primary outcomes, to generate figures commensurable with the systematic review, and the number and proportion of trials with a pre-trial protocol available online, as some editors expressed the view that protocols were more reliable.

A protocol was generated, instantiating the principles of CONSORT in simple instructions and workflows, to share with other teams who may wish to replicate this work on other journals. A full copy is posted at COMPare-trials.org. Amendments were added to this protocol as new challenges were encountered. For example, we initially planned to send corrected tables and figures to journals but this proved impractical; we reviewed our plans for ongoing monitoring after the initial six-week period; and we amended the time to publishing letters online to meet some journals’ consideration periods. All underlying data are shared online as Additional file 2 (full summary data set), Additional file 3 (full archive of all underlying raw coding sheets for each individual trial as available at www.COMPare-trials.org and template assessment sheet), Additional file 4 (COMPare protocol as at August 2016) and Additional file 5 (journal responses and themes). The correspondence archive is available at COMPare-trials.org/data.

## Results

### Workflow

We assessed 67 trials in total, a mean of 13.4 trials per journal (range 3–24). Each trial took between 1 and 7 hours to assess: workload was therefore high. One paper reported two trials, which were treated as separate trials.

### Outcome reporting quality

All forms of outcome misreporting were common; summary statistics on outcome reporting are presented in Table 1. In total, 97 primary outcomes were pre-specified in total across 67 trials (mean 1.4 outcomes per trial); of these, 76.3% were reported correctly as primary outcomes and 80.4% were reported in any form; 19.4% of trials had at least one unreported pre-specified primary outcome. The proportion of correctly reported primary outcomes varied widely between journals (range 25–96%). There were 818 pre-specified secondary outcomes (mean 12.2 per trial); of these, 55.1% were reported, and there was wide variation in reporting rates between journals (range 31–72%); 365 novel outcomes were reported without declaration in total, a mean of 5.4 per trial. Changes were rarely declared: across all trials, 13.7% of novel non-pre-specified outcomes were correctly declared as novel and non-pre-specified, as required by CONSORT. Only 29 studies had a pre-trial protocol publicly available (mean 43%, 95% confidence interval (CI) 31–56%) with journals ranging from 0 to 86%.

**Table 1:** Summary statistics on outcome reporting discrepancies

	Journal name	*Annals*	BMJ	JAMA	*Lancet*	NEJM	Total
Basic information	Number of trials included	5	3	13	24	22	67
	Journal listed as “endorsing CONSORT”	Yes	Yes	Yes	Yes	Yes	All
Protocol availability	Pre-trial protocol with pre-specified outcomes available?	0	0	7	3	19	29
	Percentage of pre-trial protocols available	0.0%	0.0%	53.9%	12.5%	86.4%	43.3%
Missing primary outcomes	Trials with any unreported primary outcomes	4	2	2	4	1	13
	Percentage of trials with any unreported primary outcomes	80.0%	66.7%	15.4%	16.7%	5.0%	19.4%
Primary outcomes	Total number of primary outcomes pre-specified	9	4	22	34	28	97
	Number of primary outcomes correctly reported as primary outcomes	4	1	18	24	27	74
	Percentage of primary outcomes correctly reported	44.4%	25.0%	81.8%	70.6%	96.4%	76.3%
	Number of primary outcomes reported anywhere	7	1	18	24	28	78
	Percentage of primary outcomes reported anywhere	77.8%	25.0%	81.8%	70.6%	100.0%	80.4%
Secondary outcomes	Total number of secondary outcomes pre-specified	49	36	111	218	404	818
	Number of secondary outcomes correctly reported as secondary outcomes	15	26	78	141	190	450
	Percentage of secondary outcomes correctly reported	30.6%	72.2%	70.3%	64.7%	47.0%	55.0%
	Number of secondary outcomes reported anywhere	15	26	78	142	190	451
	Percentage of secondary outcomes reported anywhere	30.6%	72.2%	70.2%	65.1%	47.0%	55.1%
Novel outcomes	Number of novel outcomes reported without declaration	32	25	53	192	63	365
	Mean number of novel outcomes reported without declaration, per trial	6.4	8.3	4.1	8	2.9	5.4 (95% CI 1.2–10.6)
	Percentage of novel outcomes declared as novel	5.9%	0%	39.1%	9.4%	3.1%	13.7% (95% CI 0.0–42.5%)

Abbreviations: *BMJ British Medical Journal*, *CI* confidence interval, *CONSORT* Consolidated Standards of Reporting Trials, *JAMA Journal of the American Medical Association*, *NEJM New England Journal of Medicine*

### Letter publication rates

In total, 58 trials (87%, 95% CI 78–95%) had discrepancies breaching CONSORT and therefore requiring a correction letter. Journals varied considerably as to whether trials required a correction letter (range 67–100%). All letters were submitted to journals within their submission time limit (generally within 2 weeks of trial publication). Of 58 correction letters submitted, 23 (40%) were published (95% CI 27–53%). Acceptance rates and publication delay varied widely between journals, as shown in Table 2. Two (NEJM and JAMA) rejected all letters; one (BMJ) accepted all letters as online comments only but issued a formal correction to one trial; one (*Annals*) accepted all letters online and some for print but imposed restrictions on subsequent discussion online and in the journal; and one (*Lancet*) had no facility for rapid online comments but accepted the majority (80%) of letters in print but with long delays (mean 150 days, range 40–257).

**Table 2:** Summary statistics on correction letter publication

	*Annals*	BMJ	JAMA	*Lancet*	NEJM	Total
Letters required	5	2	11	20	20	58
Percentage of letters required	100.00%	66.70%	84.60%	83.30%	90.90%	86.6% (95% CI 78.4–94.7%)
Letters published	5	2	0	16	0	23
Percentage of letters published	100%	100%	0%	80%	0%	39.7% (95% CI 27.0%–53.4%)
Mean publication delay for published letters	0 days (online)	0 days (online)	n/a	150 days	n/a	104 days (median 99 days, range 0–257 days)

Abbreviations: *BMJ British Medical Journal*, *CI* confidence interval, *CONSORT* Consolidated Standards of Reporting Trials, *JAMA Journal of the American Medical Association*, *n/a* not applicable, *NEJM New England Journal of Medicine*

### Coding amendments

For one trial (ID = 53), additional misreporting was identified after the 2-week NEJM submission deadline had passed: as all letters to NEJM were rejected, including the initial letter which identified misreporting in this trial, no further letter was sent to the journal. From feedback on all our openly shared data across all included trials, we were made aware of two outcomes that were initially miscoded. One pre-specified outcome was initially coded as unreported but was in fact given in free text in the Results section of the trial report, using very different terminology to the pre-specification text: COMPare did not require identical word matches and attempted to manually identify all outcomes reported in tables and free text; this outcome was accidentally overlooked. The second miscoded outcome was initially coded as missing but was given in the trial report, in free text, using different terminology to the pre-specification text, and was reported only in the Discussion section of the trial report. Out of 756 outcome reporting discrepancies identified by COMPare, we are therefore aware of two errors, an error rate of 0.26%. Both errors were openly acknowledged by COMPare in correspondence for journal publication.

### Themes in responses from journals

We identified a range of themes in responses from journals with respect to their understanding, and handling, of correct outcome reporting. We encountered several examples of journals expressing views that conflict with CONSORT. For example, NEJM stated that they use their judgement to decide which outcomes to report; *Annals* suggested that outcome switching is acceptable if the main results of the study are unaffected; and various editors appeared not to understand that under CONSORT it is acceptable to change outcomes as long as any changes from pre-commencement outcomes are disclosed in the paper reporting the results. We also found evidence of editors misunderstanding the importance of outcomes being pre-specified before the commencement of the trial.

Various editors made dismissive comments about registries, describing the content as unreliable or irrelevant and apparently accepting the notion that there will be multiple discrepant sets of contemporaneous pre-specified outcomes. Of note, for one trial (Trial 57, *Annals*, 03/05/16), we found three different sets of pre-specified outcomes in two registries (European Union Clinical Trials Register and ClinicalTrials.gov) and one protocol from the same time period, which is hard to reconcile with the notion of a single set of pre-specified outcomes. JAMA suggested that discrepancies between a trial report’s outcomes and pre-specified outcomes on the registry are a matter for the registry owners rather than the journal editors.

We also found editors placing responsibility for ensuring reporting fidelity onto others: NEJM suggested that editors need not ensure that reported outcomes match those pre-specified as readers can check this for themselves (although we found assessing this took between 1 and 7 hours); *The Lancet* left trialists to reply to our correspondence and did not give a view on whether editors believed that the misreported outcomes were correctly reported when asked directly.

We also encountered examples of what we coded as “rhetoric”. *Annals* made general statements about supporting the goals of COMPare. JAMA and NEJM both stated that space constraints meant that not all pre-specified outcomes could be reported, which conflicts with our finding that a mean of 5.4 novel non-pre-specified outcomes were added per manuscript. JAMA and *Annals* editors explained that they have rigorous processes to ensure that pre-commencement outcomes are correctly reported, which conflicted with our finding of extensive discrepancies and with previous work on the prevalence of outcome misreporting in the same journals. Further details and examples are presented in Table 3; a longer series of examples are presented in Additional file 5.

**Table 3:** Themes in responses from journals

Theme and subthemes	Quote	Issue
Conflicts with CONSORT		
*Failure to recognise that post-commencement changes are acceptable under CONSORT but should be declared in the paper reporting the results of the trial*	“On the basis of our long experience reviewing research articles, we have learned that pre-specified outcomes or analytic methods can be suboptimal or wrong” “Although pre-specification is important in science, it is not an altar at which to worship… [COMPare’s] assessments appear to be based on the premise that trials are or can be perfectly designed at the outset… and that any changes investigators make to a trial protocol or analytic procedures after the trial start date indicate bad science” (*Annals* Editors critique, 01/03/16).	COMPare uses CONSORT as the gold standard. CONSORT item 6b requires that trial reports declare and explain “any changes to trial outcomes after the trial commenced, with reasons” in the paper reporting the results of the trial. Changes are not forbidden; however, they should be declared in the trial report.
*Stating that outcome switching doesn’t matter if the main results of the study are unlikely to be affected*	“We reviewed materials associated with the articles and concluded that the information reported in the articles accurately represented the scientific and clinical intent detailed in the protocols... We found no inconsistencies between the audited articles and their related protocols that would justify changes in trial interpretation, corrections, or warnings to readers” (Trial 45, *Annals*, 06/04/16).	CONSORT requires all outcomes to be correctly reported; it does not distinguish between circumstances when this would, or would not, affect the overall interpretation of the intervention being trialled. It is unlikely that all outcome misreporting would change the direction or size of an overall finding; however, a culture of permissiveness around correct outcome reporting does permit misrepresentation more broadly.
*Statement describing journal practices that contradict CONSORT guidance*	“We view each piece individually and add the data as appropriate based on the judgment of the peer reviewers, the statistical reviewers, and the editors” (NEJM emails 1, 17/11/15).	CONSORT item 6b requires that trial reports declare and explain “any changes to trial outcomes after the trial commenced, with reasons” in the paper reporting the results of the trial.
*Statement that failure to report pre-specified secondary outcomes is not of interest*	“We will not ordinarily consider letters that simply... point out unpublished secondary outcomes” (JAMA emails, 09/12/15).	
*Denial of endorsing CONSORT, despite appearing on CONSORT’s list of endorsing journals*	“The New England Journal of Medicine finds some aspects of CONSORT useful but we do not, and never have, required authors to comply with CONSORT” (NEJM emails 1, 17/11/15).	
Timing of pre-specification		
*Dismissal of pre-commencement registry data as “out of date”*	“The initial trial registry data… often include outdated … entries” (*Annals* Editors critique, 01/03/16). “Registries... do not routinely monitor whether the data in the registry match the protocol, and may not be updated when the protocol changes. We therefore rely primarily on the protocol” (*Annals* Editors critique, 01/03/16).	The statement that registry data are “outdated” may reflect a broader misunderstanding about the need for outcomes to be pre-specified pre-commencement. Where the registry entry is the only accessible source of pre-specified outcomes, discrepancies should be declared as per CONSORT 6b. Even if there is a contemporaneous protocol that is not publicly accessible, the pre-specified outcomes in this protocol should match its registry entry; if not, then there are two sets of discrepant pre-specified outcomes, which requires declaration and discussion. Of note, for one trial [Trial 57, *Annals*, 03/05/16], we found three different sets of pre-specified outcomes in two registries (EUCTR and ClinicalTrials.gov) and one protocol from the same time period.
*Stating or implying that pre-specification after trial commencement is acceptable*	“We disagree with COMPare’s contention that registry data are superior to protocol information because of the timing of the former ...” (Trial 45, *Annals*, 06/04/16).	COMPare used pre-commencement outcomes from registry data only as a last resort when they were not available from a pre-commencement protocol. Pre-specification of outcomes should take place before trial commencement. CONSORT item 6b requires that trial reports declare and explain “any changes to trial outcomes after the trial commenced, with reasons” in the paper reporting the results of the trial.
Registries		
*Dismissal of registry data as unreliable*	“We check the registries, but as both authors’ responses attest, registry information can be incomplete or lack sufficient detail” (Trial 45, *Annals*, 06/04/16). “The initial trial registry data… often include... vague or erroneous entries” (*Annals* Editors critique, 01/03/16). “Registries include only extracted information” (*Annals* Editors critique, 01/03/16).	Publicly accessible trial registries are a cornerstone of trial transparency. Trialists are legally required to correctly register their trials; pre-specified outcomes are a required component under WHO guidance on trial registration; and ICMJE member journals commit to ensuring that trials are appropriately registered. Where the only source of pre-commencement outcomes contains information so imprecise that correct outcome cannot be assessed, we suggest that “inadequately pre-specified outcomes” be noted in the paper reporting the trial’s results, as this presents a similar risk of bias to misreporting of clearly pre-specified outcomes.
*Stating that discrepancies between outcomes pre-specified in a registry entry and those reported in the paper are the fault of the registry*	“Inaccuracies in the trial registration documents are more of an issue for the individuals overseeing the trial registries” (JAMA emails, 9/12/15). “We will not ordinarily consider letters that simply note discrepancies with the trial registration” (JAMA emails, 09/12/15).	It is the responsibility of the journal and trialist to ensure that a trial is correctly reported, with discrepancies against outcomes pre-specified prior to commencement declared as per CONSORT 6b. If there are discrepancies between the outcomes pre-specified and the outcomes reported in the paper, then the paper is discrepant, not the source of pre-specified outcomes. If the pre-specified outcomes on a registry are inconsistent with those in a contemporaneous protocol, then there are multiple sets of pre-specified outcomes and therefore the outcomes have not been correctly pre-specified: this should be noted in the results manuscript.
Rhetoric		
*Stating that space constraints prevent all pre-specified outcomes from being reported*	“Space constraints for articles published in the Journal do not allow for all secondary and other outcomes to be reported” (NEJM emails 1, 21/11/15).	The claim that space constraints prevent all pre-specified outcomes from being reported conflicts with the finding of COMPare, and prior research on outcome misreporting, that non-pre-specified additional outcomes were routinely added, in large numbers: a mean of 5.4 novel non-pre-specified outcomes were added per trial in COMPare (range 2.9–8.3 by journal).
	JAMA: “authors are not always required to report all secondary outcomes and all pre-specified exploratory or other outcomes in a single publication, as it is not always feasible given the length restrictions to include all outcomes in the primary report” (JAMA emails, 9/12/15).	
*General statement about supporting goals of COMPare*	“Though we share COMPare’s overarching goals to assure the validity and reporting quality of biomedical studies, we do not agree with their approach” (Trial 44, *Annals*, 15/12/16).	All such statements were accompanied by caveats, statements that explicitly or implicitly undermined the journals’ commitment to CONSORT, or incorrect statements about specific data points.
	“While the goal of the COMPare project (http://www.compare-trials.org) is noble, my colleagues and I have outlined concerns with COMPare’s approach (1)” (Trial 45, *Annals*, 06/04/16).	
Statements about journal processes		
*Statement that authors are required to declare changes to outcomes*	“When the review process generates requests for authors to report outcomes not specified in the protocol or the authors choose themselves to present such outcomes, we ask authors to indicate these as post hoc or exploratory analyses” (*Annals* Editors critique, 12/02/16).	We cannot verify whether *Annals* ask authors to do this; however, we can confirm that trials reported in *Annals* are routinely non-compliant with CONSORT, a finding which is consistent with previous research. COMPare found that, in *Annals* trials, only 6% of novel non-pre-specified outcomes added to trial reports were correctly indicated by the *Annals* manuscript as novel; a mean of 6.4 novel undeclared outcomes were added per trial; 44% of primary outcomes were correctly reported; and 31% of secondary outcomes were correctly reported.
	“To be consistent with CONSORT recommendations, we ask authors to describe, either in the manuscript or in an appendix, any major differences between the trial registry and protocol, including changes to trial endpoints or procedures” (*Annals* Editors critique, 01/03/16).	
*Statement that journal has a process to ensure correct outcome reporting*	“We carefully check for discrepancies between the protocol and the manuscript” (JAMA emails, 09/12/15).	We cannot verify JAMA’s internal processes; however, we can confirm that trials reported in JAMA are routinely non-compliant with CONSORT, a finding which is consistent with previous research. COMPare found that, in JAMA trials, 39% of novel outcomes added to trial reports were correctly indicated as novel; a mean of 4.1 novel undeclared outcomes were added per trial; only 82% of primary outcomes were correctly reported; and 70% of secondary outcomes were correctly reported.
	“We agree that it is important for researchers to pre-specify primary and secondary outcomes before conducting a trial and to report outcomes accurately in their publications. In fact, we carefully monitor this during editorial review” (JAMA emails, 9/12/15).	
Placing responsibility on others (for example, trialists or reader)		
*Stating that readers can see for themselves whether outcomes reported are discrepant with those pre-specified*	NEJM: “Any interested reader can compare the published article, the trial registration and the protocol (which was published with the article) with the reported results to view discrepancies” (NEJM emails 1, 21/11/15).	COMPare found that accessing documents and assessing trials for correct outcome reporting took between 1 and 7 hours per trial.
*Passing responsibility to trialists rather than journals or editors*	*The Lancet* published 15 out of 20 letters, mostly with accompanying responses from trialists: the majority of author responses expressed further misunderstandings about what constitutes correct outcome reporting, as reported in the accompanying paper on trialists’ responses. *The Lancet* made no comment themselves [all correspondence]. We asked the journal to clarify their position in our follow-up correspondence: “Since *The Lancet* have a longstanding positive commitment to improving reporting standards, lead the REWARD campaign on research integrity, and endorse CONSORT, we would welcome their perspective on why undeclared outcome switching in PETIT2 (and others) was apparently not addressed prior to publication; whether they now view outcome switching as acceptable; or whether they disagree that it has happened here”. We received no reply and our letter was not published (Trial 9, *Lancet*, 05/02/16).	Where a journal is listed as endorsing the CONSORT guidelines on trial reporting, it is reasonable to expect that they will take responsibility for ensuring that trials are reported consistently with these guidelines.
*Placing responsibility on trial registry staff*	“Inaccuracies in the trial registration documents are more of an issue for the individuals overseeing the trial registries” (JAMA emails, 9/12/15).	As above, if there are discrepancies between the outcomes pre-specified and the outcomes reported in the paper, then the paper, not the source of pre-specified outcomes, is discrepant.

References throughout are to the correspondence archive at COMPare-trials.org/data containing the full public correspondence on all trials, and all correspondence with editors, organised by trial ID and date, or journal name for general correspondence. Abbreviations: *COMPare* Centre for Evidence-Based Medicine Outcome Monitoring Project, *CONSORT* Consolidated Standards of Reporting Trials, *EUCTR* European Union Clinical Trials Register, *ICMJE* International Committee of Medical Journal Editors, *JAMA Journal of the American Medical Association*, *NEJM New England Journal of Medicine*, *WHO* World Health Organization

Direct engagement by editors on specific misreported outcomes was rare. NEJM did not reply to COMPare directly on this issue but shared two documents with journalists reporting on COMPare, containing what NEJM stated were errors in COMPare’s coding on two trials. For illustration, a transcript and analysis of all six NEJM responses on one trial are presented in Table 4. This demonstrates errors by NEJM editors (such as confusing outcomes timed for the fourth week *during* treatment with the fourth week *after* treatment) and also provides further examples of editors’ approach to correct outcome reporting, such as the need for time points for outcome ascertainment to be pre-specified and adhered to.

**Table 4:** Errors in *New England Journal of Medicine* responses on trial 22

NEJM quote	Issue
“[The criticism by COMPare that] AEs leading to discontinuation [were] not correctly reported... is false. Protocol indicates safety and tolerability as second of 2 primary objectives, and registration lists incidence of AEs leading to discontinuation as 1 of 2 primary outcome measures. First line of Table 3 and first sentence of Safety section (p. 2604) reports that 1 of 624 patient treated with sofosbuvir­-velpatasvir discontinued due to AE” (NEJM first comments on trial 22 (1)).	NEJM are incorrect. The outcome in question was pre-specified as a primary outcome but incorrectly reported by NEJM as a secondary outcome. COMPare therefore coded it as reported, but incorrectly reported. This is clearly denoted in the COMPare assessment sheet for this trial, and the COMPare letter reads, “There were 2 pre­specified primary outcomes, of which one is reported in the paper; while one is incorrectly reported as a secondary outcome”.
“[The criticism by COMPare that] Secondary outcome SVR [was] not reported in publication... is false. This is reported in Table 2. The COMPARE reviewers may not appreciate that SVR4 (sustained virologic response week 4) is equivalent to HCV RNA <15 IU/mL at week 4, which is reported in Table 2. HCV RNA <15 IU/mL is the lower limit of detection of the assay, as indicated in the Table footnote” (NEJM first comments on trial 22 (2)).	This is invalid. COMPare correctly coded this outcome as missing. Table 2 does report HCV RNA <15 IU/mL at “week 4” but this was week 4 during treatment (which was 12 weeks long); SVR4 is sustained virologic response at week 4 post­treatment. Hence, we correctly concluded that HCV RNA <15 IU/mL at week 4 post-treatment (SVR4) was not reported in the publication. It seems that NEJM editors did not realise that SVR4 is 4 weeks post­treatment, rather than the 4th week of treatment, hence their misunderstanding and misreporting of this outcome in NEJM and their error in their review of the letter from COMPare.
“[The criticism by COMPare that] proportion with HCV RNA < LLOQ on treatment [was] not reported in publication… is false. The COMPARE reviewer may not appreciate that “HCV RNA < LLOQ” is equivalent to “HCV RNA < 15 IU/mL”. Table 2 reports HCV RNA < 15 IU/mL during treatment” (NEJM first comments on trial 22 (4)).	This is invalid. The time point for this outcome was given in the registry entry as “up to 8 weeks”, and results were reported in NEJM only for 2 and 4 weeks. We therefore concluded that the pre-specified outcome was not reported. The fact that this discrepancy relates only to the time point is made explicit in the letter submitted by COMPare to NEJM, which states that the outcome “is not reported at the pre-specified timepoint, but is reported at two novel time-points”. Because of variation in clinical presentation over time, and the attendant risk of selective reporting, under CONSORT each separate time point at which an outcome is measured is regarded as a separate outcome.
“[The criticism by COMPare that] HCV RNA change from baseline [was] not reported in publication… is false. The change in HCV RNA from baseline is conveyed by reporting the mean HCV RNA at baseline (Table 1) and the rates of HCV RNA < 15 IU/mL (Table 2). Table S4 [of the trial report manuscript] reports the HCV RNA levels for the 2 patients who virologic failure” (NEJM first comments on trial 22 (5)).	This is invalid and represents a concerning approach to reporting pre-specified outcomes. NEJM suggests that readers calculate the results for a pre-specified outcome themselves. In addition, “HCV RNA change from baseline” cannot be calculated from the numbers reported. Mean baseline HCV RNA is reported. Mean follow-up HCV RNA is not reported. Table 2 reports only the proportion of patients with HCV RNA < LLOQ (undetectably low).
“[The criticism by COMPare that] proportion with virologic failure [was] not reported in publication… is false. This is reported in Table 2 which reports virologic failure during treatment (0 patients) and virologic failure after treatment (1 patient)” (NEJM first comments on trial 22 (6)).	This is invalid. COMPare coded this outcome as “correctly reported”. This is clear on the assessment sheet.

References throughout are to the correspondence archive at COMPare-trials.org/data containing the full public correspondence on all trials, and all correspondence with editors, organised by trial ID and date, or journal name for general correspondence. Abbreviations: *AE* adverse event, *COMPare* Centre for Evidence-Based Medicine Outcome Monitoring Project, *CONSORT* Consolidated Standards of Reporting Trials, *HCV* hepatitis C virus, *LLOQ* lower limit of quantitation, *NEJM New England Journal of Medicine*, *SVR* sustained virologic response

We also coded themes in journal editors’ criticisms of the letter-writing project. The dominant theme was misrepresentation of COMPare’s technical approach. For example, *Annals* editors stated that COMPare’s protocol was unreasonable because it required exact word matches between pre-specified and reported outcomes (it does not) and that COMPare only used registries as a source of pre-specified outcomes (as per the COMPare protocol, registries were used as a last resort when no pre-commencement protocol was available). JAMA stated that COMPare’s responses to published trial reports contained insufficient information (all COMPare raw data sheets were shared in full, detailing each pre-specified primary and secondary outcome; whether and how each pre-specified outcome was reported; each additional non-pre-specified outcome reported; and whether each non-pre-specified outcome added was correctly declared as non-pre-specified). It cannot be ascertained whether these inaccuracies represent misunderstandings or acts of rhetoric. Further details are presented in Table 5 and Additional file 5.

**Table 5:** Themes in journals’ criticisms of COMPare

Theme	Quote	Issue
*Misrepresentation of COMPare’s methods*	COMPare’s method is a “simple check for an exact word match between outcomes entered in a registry and those reported in a manuscript, but that oversimplifies a highly nuanced process” (*Annals* to BMJ).	This is untrue. COMPare did not seek literal word matches: each pre-specified outcome was manually checked and re-checked, as per previous research on outcome misreporting, using CONSORT as gold standard.
	“The initial trial registry data… serve as COMPare’s ‘gold standard’” (*Annals* Editors critique, 01/03/16).	This is untrue. As explained in our publicly accessible protocol, COMPare used the registry entry only as a last resort where there was no pre-commencement protocol publicly available, as CONSORT 6b requires that changes after commencement be noted in the trial report. Notably, no *Annals* trial had a publicly accessible pre-commencement protocol.
*Stating that COMPare correspondence and raw data sheets contained insufficient information*	“In addition, some of the information in your letters is vague, containing only numbers and not specific outcomes, making it difficult to understand the specific issues or reply to them. Moreover, the last 2 paragraphs of the letters you have submitted, concerning CONSORT and the COMPare project, are identical” (JAMA emails, 09/12/15).	All correction letters linked to the COMPare online repository where all underlying raw data sheets were shared in full, specifying in detail each pre-specified primary and secondary outcome, whether and how each pre-specified outcome was reported, each additional non-pre-specified outcome reported, and whether each non-pre-specified outcome added was correctly declared as non-pre-specified. This JAMA letter was received halfway through the COMPare study period. To address the reasons given for letter rejection, despite word length limits imposed by JAMA for correspondence, all subsequent JAMA letters had no repetition and extensive detail within the text on specific misreported outcomes. However, none of these subsequent letters was published and we received no further replies.
*Warning readers against COMPare’s assessments*	“Until the COMPare Project’s methodology is modified to provide a more accurate, complete and nuanced evaluation of published trial reports, we caution readers and the research community against considering COMPare’s assessments as an accurate reflection of the quality of the conduct or reporting of clinical trials” (*Annals* Editors critique, 01/03/16), (Trial 25, *Annals*, 14/12/15), (Trial 44, *Annals*, 15/12/16), (Trial 45, *Annals*, 15/12/15), (Trial 68, *Annals*, 30/12/15).	All *Annals*’ critiques on matters of fact were incorrect; *Annals* rejected replies demonstrating this to readers. Following this comment posted by *Annals* under all COMPare correspondence, no trialists engaged with any of our evidence of their failure to correctly report pre-specified outcomes. We regarded *Annals*’ advising authors not to engage with reasonable professional criticism of their methods and results as a breach of ICMJE guidance on correspondence.
*Claim that COMPare coding incorrect on specific outcomes*	NEJM gave journalists a detailed review of COMPare’s assessment of one trial, which NEJM stated had identified six errors in COMPare’s assessment. This was reviewed, and NEJM were wrong on all six counts; full details are presented in the table above and in the correspondence appendix (NEJM first comments on trial 22). Another NEJM review of a COMPare letter was also factually wrong on all three issues it raised (NEJM second comments on trial 22 (2)).	The editors were wrong on all nine issues raised. The document they sent exemplified misunderstandings around the importance of reporting all pre-specified time points for each pre-specified outcome.

References throughout are to the correspondence archive at COMPare-trials.org/data containing the full public correspondence on all trials, and all correspondence with editors, organised by trial ID and date, or journal name for general correspondence. Abbreviations: *BMJ British Medical Journal*, *COMPare* Centre for Evidence-Based Medicine Outcome Monitoring Project, *CONSORT* Consolidated Standards of Reporting Trials, *ICMJE* International Committee of Medical Journal Editors, *JAMA Journal of the American Medical Association*, *NEJM New England Journal of Medicine*

We also found some positive responses. The BMJ issued a 149-word correction on the REEACT (Randomised Evaluation of the Effectiveness and Acceptability of Computerised Therapy) trial after receiving COMPare’s correction letter, and *Annals* corrected the “Reproducible Research” data-sharing statement on one trial after we reported that a protocol was withheld from us by the trialists. No other formal corrections were issued on any of the 58 misreported trials.

### Narrative account of individual journals’ responses

Journals’ responses to correction letters reporting breaches of CONSORT were diverse but broadly dismissive. NEJM rejected all COMPare letters, stating “we do not, and never have, required authors to comply with CONSORT” and explaining that “space constraints” prevent all outcomes from being reported [NEJM emails 1]. COMPare appealed and received no reply. In March, NEJM gave journalists a detailed review of COMPare’s assessment of one trial, which NEJM stated had identified six errors in COMPare’s assessment, as per Table 4 above.

JAMA published no letters and informed us halfway through the project that they would publish none, as in their view COMPare letters contained repetition and too little information on specific misreported outcomes [JAMA emails, 09/12/15]. JAMA imposes word length restrictions on letters responding to papers, and this limit prevented us including all details of all misreported outcomes, however all letters signposted COMPare-trials.org where all underlying raw data were shared in full. For all letters submitted to JAMA after their December 9 reply, we removed all repetition and added specific detail of every misreported outcome in the main body of the text. None of these letters was published, and we received no further correspondence from JAMA. Of note, JAMA also stated “trial protocols ... have been included as a supplement with each trial published in JAMA since mid-2014”. We found that pre-commencement protocols were available for only 53.9% of JAMA trials in our cohort.

*The Lancet* published 16 out of 20 COMPare letters, mostly with author replies. Most author replies contained misunderstandings of correct pre-specification and reporting of outcomes, as reported in our accompanying paper on trialists’ responses. We sent several replies addressing these issues and two of them were published. Several of these replies requested that *Lancet* editors express a view on whether outcomes had been correctly reported. We have received no comment from *The Lancet* editors throughout. The BMJ published only three trials during the study period but published all COMPare correspondence online; they issued a formal correction for one trial but not for another which had similarly misreported outcomes. *Annals* engaged in a lengthy and complex dispute with COMPare, as detailed in the timeline in Table 6.

**Table 6:** Timeline of *Annals*’ responses to COMPare

October–December 2015: Following submission, all COMPare letters were accepted as online comments only. Reading these requires registration for an *Annals* user account.	
14 December 2015: *Annals* editors published an 850-word critique of COMPare as an online comment on the CASCADE trial, which later appeared as a full page article in print in the March 1 edition and as a standalone online letter. This piece has no named authors. It contained various incorrect and internally inconsistent statements on outcome pre-specification and reporting, as documented in Table 3 and online [20]. *Annals* declined to publish a response from COMPare in print or below the standalone online letter (Trial 5, *Annals*, 14/12/15). In their critique, *Annals* stated that they prefer to use protocols over trial registries and that registries often contain “outdated, vague or erroneous entries” (Table 3). However, pre-trial protocols were not available for any of the *Annals* trials assessed by COMPare. From February to April 2016, the official *Annals* social media account claimed, incorrectly, to have fully published COMPare correspondence on four occasions [*Annals* tweets] after their non-publication of COMPare responses was reported elsewhere [21].	
14–30 December 2015: *Annals* editors posted an identical comment beneath four out of five COMPare comments on misreported trials in *Annals*: “we caution readers and the research community against considering COMPare’s assessments as an accurate reflection of the quality of the conduct or reporting of clinical trials... we do not believe that COMPare’s comments on [trial] merit a response”. In our view, this conflicts with ICMJE guidelines: “The authors of articles discussed in correspondence ... have a responsibility to respond to substantial criticisms of their work using those same mechanisms and should be asked by editors to respond” [22]. Following this comment from *Annals* editors, no authors engaged on the issue of whether they had reported their pre-specified outcomes. In March 2016, *Annals* clarified their comments, asserting that their comment was not intended to dissuade authors from replying to COMPare comments. No trial authors have replied on the concerns raised about their outcome reporting since *Annals*’ initial comment.	
1 March 2016: Two COMPare comments were published in print in *Annals*, with responses from an author and the editors (mentioned above). Both of these responses contained further errors and misunderstandings in relation to the outcomes published in the trial report; *Annals* declined to publish subsequent COMPare correspondence pointing out these issues.	
18 March 2016: Following *Annals* editors stating that protocols should be used to assess outcome reporting fidelity in preference to registry entries, COMPare requested the protocol for Trial 45 (Everson *et al*.) from the lead author: this protocol was not published, but the “Reproducible Research Statement” in *Annals* stated that it was available on request. We received a reply from Gilead sciences, stating that the protocol is confidential [Everson emails, *Annals*]. COMPare raised concerns about this in a further online comment to *Annals* [Trial 45, *Annals*, 19/04/16]; *Annals* subsequently issued a correction to the Reproducible Research Statement.	
19 April 2016: *Annals* changed its “Instructions to Authors” to require submission of protocol for subsequent publication alongside all trials in the future. *Annals* told journalists that this change was planned and predated COMPare’s concerns [23].	

References throughout are to COMPare-trials.org/data, containing the full correspondence on all trials, organized by trials ID and date, or journal name for general correspondence. Abbreviations: *CASCADE* Clopidogrel After Surgery for Coronary Artery Disease, *COMPare* Centre for Evidence-Based Medicine Outcome Monitoring Project, *ICMJE* International Committee of Medical Journal Editors

## Discussion

### Summary

We found that journals listed as endorsing the CONSORT guidelines, which require complete outcome reporting, fail to ensure compliance on this issue. The majority of correction letters were rejected. In addition, we found that two journals actively rejected all letters that signposted outcome misreporting, despite its being an important source of bias; and several journals disclosed that, contrary to their being listed as endorsing CONSORT, they do not regard breaches of CONSORT as problematic. Qualitative analysis of themes in extensive subsequent correspondence with journal editors and trialists demonstrates widespread misunderstandings of what constitutes complete outcome reporting. We additionally found breaches of best practice policies such as ICMJE guidelines.

### Strengths and weaknesses

Post-publication peer review is an important component of the scientific process. There have been previous anecdotal reports of shortcomings around how journals handle individual items of critical correspondence [12, 13]: however, to the best of our knowledge, COMPare is the first systematic and prospective study setting out to generate and submit a comparable cohort of correction letters on a large systematic sample of misreported scientific studies, in order to assess how scientific journals are curating critical post-publication peer review. It is also the first to systematically assess whether journals will permit open discussion of possible editorial shortcomings.

The key strength and innovation of our study is that it was conducted prospectively, aiming to correct individual misreported trials in real time rather than retrospectively publishing an overall prevalence figure. This allowed us to go beyond previous work that documents only prevalence and instead generate data shedding light on the reasons for misreporting and, through letter acceptance rates, also generate an objective measure of journals’ commitment to correct reporting.

Our novel approach of prospective real-time corrections brought several additional methodological benefits. Previous work has mostly assessed whether there was outcome switching at all in a study [1]. To maximise the informativeness, credibility and impact of COMPare’s letters, we needed to assess the extent of outcome misreporting in more detail and share information on each individual misreported outcome. Because of this, and a broader commitment to open science in the team, all underlying raw data were shared in full during the project and prominently signposted in journals and all external coverage. This open approach is likely to have reduced the risk of small coding errors: our data were closely scrutinised by trialists and editors motivated to find evidence of errors; and we were highly motivated to ensure that no errors were found. None of the 29 previous cohort studies has shared data on individual outcomes and trials in this fashion. From all trialist feedback, we found two outcomes miscoded by COMPare out of 756 identified outcome discrepancies; no assertions of miscoding made by editors were valid (see Table 2 and Additional file 5 for examples). It remains possible that our data set contains small additional coding errors, as with all research. However, our prevalence figures are consistent with previous work, and at least three members of the research team reviewed each outcome.

An additional strength of our study is the lack of conflict of interest among the COMPare researchers on the specific interventions being trialled. Critical correspondence on methodological shortcomings in published research often originates from other academics in the same field who may have a personal history, financial or ideological conflicts of interest, or a competitive relationship with the individual research teams involved: this was not the case for our large systematic sample of trials and letters. However, we note that the COMPare team do have a complex range of additional conflicts of interest, in excess of what would normally be declared; for example, an academic in our position may be concerned not to appear critical of a journal, given the importance of journal publication to career progression; all senior academics on the team have previously published in at least one of the journals covered, and two of us (BG and CH) have previously worked with the BMJ on a transparency campaign.

The issue of generalisability is key. Our study covered all trials in five general medical journals, reporting a wide range of interventions from hand washing and acupuncture to antiviral drugs. However, all journals were very high-impact: lower-impact journals may have different or more heterogeneous performance on outcome reporting and publication of correction letters. Furthermore, the fact that our letters were part of a coordinated project may have led editors to treat them differently: it is hard to ascertain whether this would make editors more or less likely to handle them appropriately.

Ideally, our study would have examined trials from a wider sample of journals. However, the workload associated with checking trials in real time, in detail, and maintaining subsequent interactive correspondence within the timeline for publication was extremely high, even for a large coordinated team; and the intention of COMPare was not solely to measure the prevalence of outcome misreporting. Initial plans to maintain the process of analysing trials and submitting correction letters were shelved, due to the high workload and blanket rejection of most letters.

### Context of previous work

Our findings on the simple prevalence of outcome misreporting are consistent with previous work. The most current systematic review, from 2015 [1], found 27 studies comparing pre-specified outcomes against those reported, as described above: the median proportion of trials with a discrepancy on primary outcomes was 31% (range 0–100%, IQR 17–45%); in COMPare, we found that 19.4% of trials (95% CI 9.9–28.9%) had unreported primary outcomes. Therefore, while some journals argued that our assessment process was unreasonable, COMPare found a lower prevalence for discrepancies than previous work. Although most previous studies were published within the past decade, all but one included trials that commenced before the ICMJE 2005 policy mandating trial registration with pre-specified outcomes as a condition of acceptance for publication in member journals: this may have led to improved reporting standards. However, our findings give little evidence for any strong overall improvement, and two additional cohort studies published in 2008 [14] and 2016 [15] report similar prevalence for discrepancies as before.

All previous studies have published only prevalence figures describing the overall extent of outcome misreporting, and none attempted to actively correct the record on individual misreported trials. It is widely agreed that the scientific literature should be self-correcting, with researchers engaging in post-publication peer review and submitting critical commentary or corrections in letters for publication, sometimes resulting in formal corrections or retraction where the main results of a study are invalidated by an error identified. Prior to our study, there have been only anecdotal reports that these systems fall short when tested. The largest we are aware of is a retrospective narrative description of four academics’ experience attempting to publish correction letters on 25 studies with various flaws that they identified while writing a newsletter for their research field: they found that “post-publication peer review is not consistent, smooth, or rapid” with journal editors unwilling to publish critical letters, correct errors, or retract articles with errors that invalidate their key findings [13].

The current Cochrane review of studies examining discrepancies between protocols or registry entries and published trial reports [16] reports high prevalence for numerous other related reporting flaws, including inconsistencies on sample size calculation, blinding, method of allocation concealment, subgroup analyses, and analytic approach. It is therefore highly likely that the problems we have identified with misreporting, and failure to correct that misreporting, will generalise beyond the single issue of outcome reporting.

### Policy implications for journals

Some journals explicitly stated that they did not expect all pre-specified outcomes to be correctly reported, despite being publicly listed as endorsing the CONSORT guidelines which state the contrary. This disparity between public stance and editorial practice is likely to give false reassurance to readers, who may reasonably assume that all pre-specified outcomes are correctly reported in a journal article. While CONSORT compliance would be preferable, we suggest as a minimum that all medical journals explicitly clarify whether they do, or do not, aim to comply with CONSORT (if so, which elements) and specify the documents and methods they use to assess compliance. The workload for our team of independently checking for outcome misreporting was extremely high: however, this would be lower for journals, as any apparent discrepancy could be referred back to the authors, whereas for COMPare letters, an extremely high level of confidence in discrepancies—and therefore more laborious checking—was required before submission.

Most letters were not published, and we encountered instances of what we regard as a failure to abide by best practice around journal correspondence as set out in ICMJE guidance (Table 3, *Annals*). Since journal editors make editorial judgements about what is published in their journals, they may have significant conflicts of interest when their own editorial processes or judgements are subjected to critical scrutiny. *The Lancet* has an internal ombudsman issuing an annual report [17]. There have been calls for independent oversight of journal editors for many years [18]: although an external appeals process would likely be valuable, it also risks being cumbersome or vulnerable to abuse by special interest groups. We are aware of no evidence assessing the effectiveness of this approach. At a minimum, we suggest that all correspondence above a basic minimum threshold for quality be published accessibly online, as per Rapid Responses in the BMJ.

We also identified asymmetries in access to critical post-publication peer review. For example, at *Annals*’ website, all visitors (including those with no password or subscription) can read an abstract that misreports a trial’s outcomes, but only those with password-controlled registration and account access can read online comments demonstrating that pre-specified outcomes were misreported. In addition, there are restrictions on critical post-publication correspondence that may not be justifiable in the era of online publication. For example, most journals had length limits and tight submission deadlines for letters: both of these have been previously criticised [19]. There is further asymmetry here: *The Lancet* gives readers 2 weeks to submit correspondence on a specific paper yet did not publish some COMPare letters until more than 6 months after initial trial publication. During this period—possibly the period when the trial reports were most read—information about outcome misreporting was effectively withheld from readers. Overall, our findings suggest that post-publication peer review and critical appraisal are not currently well managed by journals. This suggests that alternative approaches such as PubMed Commons—with a lower threshold, instant online publication, good indexing, and independent editorial control—may be more appropriate.

### Policy implications for registries

The denigration of trial registry data by some editors was unexpected. Registries were specifically set up as a public time-stamped information resource to address selective outcome reporting. They have received extensive public support from WHO, journals, and ICMJE, who state: “The purpose of clinical trial registration is to prevent selective publication and selective reporting of research outcomes”. The key driver for greater registry uptake was action by journals and specifically the ICMJE stating in 2005 that member journals would not consider unregistered trials for publication [7]. Journals taking the content of registry entries seriously would therefore likely be a key lever in ensuring that registries are used appropriately by trialists; that would be valuable, as we found that registries are often the only publicly accessible time-stamped source for a trial’s pre-specified outcomes. There are no valid reasons why registries should contain outcomes that are discrepant with contemporaneous protocols, as some argued in response to COMPare letters; indeed, trialists in many territories, including the US and European Union, have a legal duty to completely and accurately register their trial, including details of pre-specified outcomes, on a register with a statutory regulatory role. Despite this, for one trial, we found three different contemporaneous sets of pre-specified outcomes spread across two registry entries and one protocol.

Journals varied widely in their practical approach to registries. For example, the BMJ uses registries as the primary source for pre-specified outcomes, whereas *Annals* editors told COMPare that protocols were their chosen source for assessing complete outcome reporting, but none of five trials published in *Annals* had a pre-specified protocol publicly available. *Annals* policy and practice therefore make independent verification of their assessment of correct outcome reporting impossible. We see no justification for relying on protocols when they are routinely unavailable and when registry entries—a legal requirement on publicly accessible services explicitly set up to address selective reporting—are now almost universally available. We hope that trial registry managers will also find our data on some editors’ approaches to their work informative in their broader strategic approach.

### Policy implications for CONSORT

We believe that there is a need for greater clarity, emphasis, and awareness raising on certain aspects of CONSORT guidance and a need to review the mechanisms around the EQUATOR (Enhancing the Quality and Transparency Of health Research) network’s public list of journals “endorsing” CONSORT. Since some journals we examined eventually stated that they do not require CONSORT compliance on the key issue of correct outcome reporting, CONSORT may wish to consider removing journal titles from their list, or implementing a two-level approach, with journals opting to “endorse” in spirit or “enforce” in practice, or possibly consider offering a system to check and accredit compliance, for journals wishing to demonstrate credibility to readers.

### Future work

There are already extensive data on the simple prevalence of methodological and reporting errors for clinical trials. In our view, there is little value in repeating simple prevalence studies unless there are grounds to believe that the prevalence has changed. While we recognise that other research teams may be intimidated by the response our project received, the mixed methods approach of COMPare provides additional insights into the reasons why shortcomings persist despite public statements of adherence to reporting standards.

It is plausible that the modest coverage, impact, internal discussions and public debate triggered by our systematic programme of corrections have had a positive impact on policy or practice at journals. We are therefore now re-assessing outcome reporting in the same five journals to assess whether standards have improved following the initial COMPare study and feedback period. We would welcome others repurposing our methods and have shared our protocol in full online and as Supplementary Material, expanded where appropriate to clarify specific steps for those unfamiliar with specific requirements of CONSORT. We hope other groups may find this useful to run a similar project in a different set of specialty journals, the same journals, or other sectors where RCTs are becoming commonplace, such as development economics, education, or policing. Our method could also be extended to other methodological and reporting issues, including in fields outside of medicine, especially where there are similar methodological shortcomings that can be identified consistently, to produce a similarly comparable cohort of letters. This would allow researchers to assess whether the problem of journals rejecting legitimate critical commentary is limited to high-impact medical journals with a clinical focus and would move current high-profile discussion on shortcomings at journals forward from anecdotal descriptions of challenges around criticising individual studies.

In our view, the traditional model for research on shortcomings in studies’ methods and reporting—publishing prevalence figures alone, for retrospective cohorts—represents a wasteful use of resources. Specifically, it is a waste of the insights generated by expert reviewers, at considerable time and expense, about shortcomings in individual studies. We suggest that all such studies systematically write letters for publication about each individual misreported or flawed study they identify, in order to alert consumers of the academic literature to those flaws, to maximise efficient use of researcher time, to raise awareness of methodological flaws in published research, and to augment the impact of their work. This simple change will help academia to be a learning system with constructive feedback. In addition, it is likely to improve the data quality in methodological research, for the reasons described above, as researchers of studies coded as flawed will be able to openly contest adjudications they regard as inaccurate.

## Conclusion

We found high levels of outcome misreporting amongst the five top medical journals listed as endorsing the CONSORT statement on correct reporting of clinical trials. Most of these journals rejected correction letters that documented their misreporting. We found extensive evidence of misunderstandings about correct outcome reporting at journals. The disparity between journals’ public stance and practical action may mislead readers into assuming that pre-specified outcomes are correctly reported. Possible solutions include changes to correspondence processes at journals, alternatives for indexed post-publication peer review, changes to CONSORT’s mechanisms for enforcement, and changes to traditional practices in methodology research to ensure that problems identified with published studies are routinely shared with the broader academic community.

## Additional files

Additional file 1: a General template letter. b Template letter to the *New England Journal of Medicine* (NEJM). c Template letter to *The Lancet*. (ZIP 20 kb)

Additional file 2: Full summary data set. (XLSX 58 kb)

Additional file 3: Full archive of all underlying raw coding sheets for each individual trial as available at www.COMPare-trials.org. (DOCX 6 kb)

Additional file 4: COMPare current protocol as at August 2016. (DOCX 93 kb)

Additional file 5 Journal responses and themes. (PDF 96 kb)

## Abbreviations

BMJ: *British Medical Journal*
CI: Confidence interval
COMPare: Centre for Evidence-Based Medicine Outcome Monitoring Project
CONSORT: Consolidated Standards of Reporting Trials
ICMJE: International Committee of Medical Journal Editors
IQR: Interquartile range
JAMA: *Journal of the American Medical Association*
NEJM: *New England Journal of Medicine*
RCT: Randomised controlled trial
WHO: World Health Organization
