# Supplementary material for: COMPare: a prospective cohort study correcting and monitoring 58 misreported trials in real time
Source: Trials. 2019 Feb 14;20:118. doi: 10.1186/s13063-019-3173-2 (PMC6375128; doi:10.1186/s13063-019-3173-2)
Supplement: Supplementary file 3 — Full archive of all underlying raw coding sheets for each individual trial as available at www.COMPare-trials.org. (DOCX 6 kb) [file 13063_2019_3173_MOESM3_ESM.docx]

Full archive of raw assessment sheets available at COMPare-trials.org. We are happy to submit an additional appendix containing a copy of all raw assessment sheets if prefered.
