# Supplementary material for: COMPare: a prospective cohort study correcting and monitoring 58 misreported trials in real time
Source: Trials. 2019 Feb 14;20:118. doi: 10.1186/s13063-019-3173-2 (PMC6375128; doi:10.1186/s13063-019-3173-2)
Supplement: Supplementary file 4 — COMPare current protocol as at August 2016. (DOCX 93 kb) [file 13063_2019_3173_MOESM4_ESM.docx]

The Centre for Evidence Based Medicine Outcome

Monitoring Project (COMPare)

Protocol

Ben Goldacre, Henry Drysdale, Carl Heneghan

CEBM, University of Oxford

[compare-team@ebmdatalab.net](mailto:compare-team@ebmdatalab.net)

*Last updated: 18/05/2016. Full details of amendments, dates and reasons are given in appendix 1*

**Background**

Despite various efforts, academic journal reports on clinical trials continue to exhibit outcome reporting bias: pre-specified primary and secondary outcomes are left unreported, while non-pre-specified outcomes are reported as main results without any clear declaration to that effect. While there have been various publications setting out the prevalence of this problem, there has been no systematic effort to publicly identify the individual trials and journals that have engaged with this form of misreporting. There are good grounds to believe that this problem may become less prevalent through a process of individual accountability, and transparent access to good quality information on which individual researchers and journals are currently engaging in this practice.

We aim to implement such a monitoring system, to check whether academic journals are able to manage corrections to the record, and to reduce the prevalence of the problem. Our specific objectives are to:

1. Determine the proportion of pre-specified outcomes that are correctly reported in the top 5 general medical journals.
2. Determine the prevalence of undeclared, non-pre-specified outcomes reported in these journals.
3. Submit letters to journals setting out the discrepancies, in order to correct the record, and assess whether these are published at all, and in a timely fashion.
4. Share our results openly to increase awareness of the presence of misreported outcomes in specific trials and journals.

**Methods**

We will assemble a group of students and researchers able to work on the project (assessors), with one study coordinator to administer sharing out trials.

We will create an assessment template, a letter template, and a master tracker, as Google spreadsheets.

Each week the study coordinator will screen the top 5 medical journals (based on impact factor) to identify all trials, enter the brief details of these onto the tracker, and assign each trial to an assessor and second assessor.

Each assessor will carry out the steps below for each trial.

**The assessment system**

1. Use the assessment template to make a new assessment sheet for the trial (with study ID appended in title) and save in the study Google drive folder.
2. Check to see if there is a version of the trial protocol available that is dated before the trial start date. If so, this should be used to gather pre-specified outcomes (point 5).
3. Find the trial’s registry entry, using clinicaltrials.gov or the WHO clinical trials search portal http://apps.who.int/trialsearch/. Copy the link to the online registry to the master tracker and assessment sheet.
4. Save in the folder for that trial:
   1. pdf of trial paper.
   2. Any appendices or supplementary material.
   3. pdf of pre-trial protocol, if available.
5. Copy the pre-specified primary and secondary outcomes from either the pre-trial protocol or registry entry into the assessment sheet.

Note:

- The trial protocol should only be used for pre-specified outcomes if it was published before the trial start date.
- If using the registry entry, any changes made after the trial start date will be discounted. The pre-specified outcomes will be taken from the registry entry as it was immediately before the trial start date.
- If both the registry entry and protocol were published before the trial start date, but the protocol does not sufficiently define the pre-specified outcomes, the registry entry will be used instead.

1. Select `yes', `no', or `unclear' from the drop-down menus next to each outcome to show if it is:
   1. reported in main trial results table
   2. reported somewhere in the paper other than the main results table (e.g. free text, appendix)
   3. not reported in the paper at all.
   4. not sufficiently defined as outcome to know whether or not it was reported.Note: If ‘unclear', details should be given in a `comment' attached to the relevant cell in the spreadsheet.
2. List all non-pre-specified outcomes reported in main results table in the assessment sheet.
3. Select `yes' or `no' in the box next to each of these outcomes to show if it was correctly declared as non-pre-specified in the report.
4. Enter basic results from the assessment into the `Master Tracker' spreadsheet.

Each assessment will then be independently checked by a randomly assigned second assessor. Any discrepancies will be recorded in the master tracker as present (`yes' or `no'), and with free text details in the next column. A solution to these discrepancies will be decided upon by discussion with the COMPare team at the weekly meeting.

Following this, the assessor will write a letter to the journal, for each trial where there are discrepancies between the registry and the publication. This will be done using a standard template letter which will explain the motivations and aims of the project, and then summarise the problems found with the trial in question. There is a specific template letter for each journal, to reflect the different correspondence requirements.

Where it adds to the content of the letter, we will also add **either** Table 1 **or** Table 2, which will be produced by the assessor.

**Table 1** will be an annotated version of the trial’s main results table, showing: which reported outcomes were pre-specified primary outcomes (by writing **P1** to the right of the table); which were pre-specified secondary outcomes (by writing **P2** to the right of the table); and which were not pre-specified at all (by writing **U** to the right of the table).

**Table 2** will be a corrected results table for the trial that lists each pre-specified primary and secondary outcome; gives the results for each (if they are available in the trial publication, or registry results page); or reports them as `missing' if those values are not available.

These letters will be sent for publication to the journal editor by the study team on a weekly basis. We will publish a description of the reporting errors in each trial on the COMPare website, with read-only links to the assessment and letter for that trial, in the following situations:

1. If the letter for that trial is published by the journal.

2. If the letter for that trial is rejected by the journal.

3. If four weeks have passed without publication: this is four weeks from letter submission; or, if the journal specifies a time period for editorial letter review of eight weeks or less after publication (with a letter submission deadline of four weeks from publication), then this time period.

4. If the standard of outcome reporting was high and a letter was not required.

In these situations, we will also post our letters on PubMed Commons.

On the COMPare-trials.org website, individual trials’ data will be obfuscated until conditions 1-4 above are met, but overall figures on outcome reporting will be produced that include all data from all trials coded to date.

Where responses to letters are received from trial authors or journal editors, a reply will be generated by discussion with the COMPare team.

**Analysis**

We will conduct an omnibus analysis of all data collected during the first 6 weeks of the project, and submit this for publication, alongside a description of our aims and methods.

Primary Outcomes:

1. Proportion of pre-specified outcomes reported.

2. Proportion of reported outcomes that are non-prespecified, and not declared as such.

3. Proportion of letters published in print.

4. Publication delay in days.

Secondary Outcomes:

1. All the above outcomes, broken down by journal.

We hope to continue monitoring outcome switching, in order to complete our audit loop, following publication of the results of our initial analysis phase.

**An example of annotating a trial results table:**


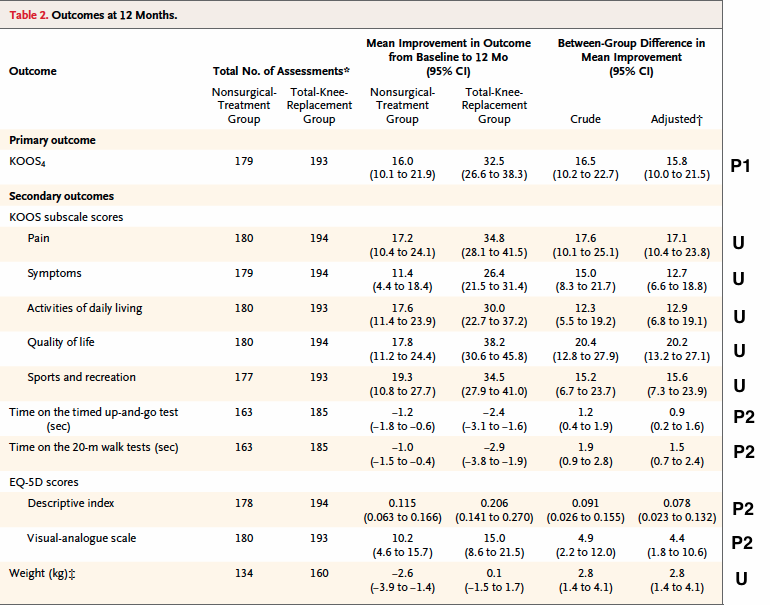


**Appendix 1: Updates and changes to our protocol since start of COMPare analysis**

This protocol is principally shared as an operations manual, so that others can use our method to run the same project in specialty journals: we hope it is useful. Protocol amendments made during the project are described below. Amendments are generally to operationalise the principles of CONSORT, and disambiguate what to do in practical situations that arose during the project, producing a clear pathway for others.

1. 29/11/2015: Small modifications to specifics of “objectives” since first draft in pilot stage. Removal of “reduce prevalence of misreported outcomes” and addition of “submit letters”. This is simply a clarification of objectives: the letter submission process has been part of the protocol in the “assessment system” section since the start of the project.
2. 29/11/2015: Addition of Appendix 2, as we iteratively develop an additional taxonomy of *types* of poor reporting and poor prespecification from working with the registry entries, protocols, and publications during the project. This is a clarification of what constitutes good outcome prespecification and reporting, consistent with the CONSORT guidelines.
3. 04/12/2015: Modification of letter templates during october and november: slight differences between letter templates for submission to each journal, to reflect different journals’ submission requirements and restrictions on word length. Decided that Table 1 and Table 2 (described above) should be optional additions to the letters, used only where they add to the content of the letter.
4. 04/12/2015: Addition of our plan for ongoing monitoring, and stopping / sampling rules for this, if person-time permits.
5. 04/12/2015: Addition of explicit mention of regular weekly meeting with team (often twice weekly due to workload).
6. 04/12/2015: Addition of rules around time limits for posting results to COMPare-trials.org (and calculating rolling summary statistics) as website developed during early phase of project.
7. 7/12/2015: Specified simple analysis for omnibus paper
8. 7/12/2015: Added team email address, compare-team@ebmdatalab.net
9. 7/12/2015 - amended start date for 4 week delay before publicly sharing letter and data. This is because we established that some journals (e.g. JAMA) consider letters for publication for a set period of time after their publication date. Since there was little material difference between this time period (8 weeks) and “4 weeks after submission” (with submission deadline of 4 weeks after publication) we felt that our time limit for letter publication should reflect this, in order to work within the system to correct the record on misreported trials.
10. 7/12/2015 - added note to assessment system “If both the registry entry and protocol were published before the trial start date, but the protocol does not sufficiently define the pre-specified outcomes, the registry entry will be used instead.” We added this because, although our policy initially assumed protocols were superior and more detailed, we have found that outcomes are sometimes insufficiently defined in the protocol and defined more completely in the registry entry. We discuss an example elsewhere.
11. 18/05/2016 - Addition of “since start of COMPare analysis” to title of appendix 1, for clarification.
12. 18/05/2016 - Addition of dates and reasons for changes to points 1-10 above, in the interests of transparently sharing our workflow.
13. 18/05/2016: changed plans for continuous monitoring to after publication of our omnibus analysis paper. Due to the workload of responding to journal editors and trial authors, continuous monitoring was not possible, and has been deferred until after publication of our first set of papers on the project.

**Appendix 2: Correct pre-specification and correct reporting of outcomes**

All primary and secondary outcomes in a clinical trial must be fully pre-specified on a clinical trials registry and in the trial protocol before the trial start date. We consider correct pre-specification of an outcome to contain details of:

1. The variable that will be measured
2. The specific method of measurement
3. The time-points at which the outcome will be reported

Some specific points regarding pre-specified outcomes:

- Where an outcome has been pre-specified at several time-points, each time-point is considered to be a separate outcome.
- Where scoring systems are used that contain several sub-scales, it must be pre-specified whether the outcome consists of the overall score or specific sub-scales.
- In general, an outcome must be unambiguously pre-specified, so that the reader can be sure if the outcome was reported or not.

Correct reporting of outcomes:

1. All pre-specified outcomes must be reported in the published trial report.
2. Primary outcomes must be clearly reported as the main results of a trial. This is usually in the main trial results table, however other clear methods of reporting, such as Kaplan-Meier plots, are also acceptable.
3. Secondary outcomes should be clearly reported as such. At a minimum, this should be in the free text results section of the trial report. Secondary outcomes must not be reported as primary outcomes.
4. Any discrepancies between the outcomes pre-specified and those reported must be clearly declared in the trial report, along with details of the timing of changes and reasons for the discrepancies.
