# Supplementary material for: COMPare: a prospective cohort study correcting and monitoring 58 misreported trials in real time
Source: Trials. 2019 Feb 14;20:118. doi: 10.1186/s13063-019-3173-2 (PMC6375128; doi:10.1186/s13063-019-3173-2)
Supplement: Supplementary file 5 — Journal responses and themes. (PDF 96 kb) [file 13063_2019_3173_MOESM5_ESM.pdf]

Appendix. Themes of responses from journals.

Theme and Subthemes	Quote	Issue
**Conflicts with CONSORT**		
Failiure to recognise that post-commencement changes are acceptable under CONSORT, but should be declared in the paper reporting the results of the trial.	“On the basis of our long experience reviewing research articles, we have learned that prespecified outcomes or analytic methods can be suboptimal or wrong” “Although prespecification is important in science, it is not an altar at which to worship… [COMPare’s] assessments appear to be based on the premise that trials are or can be perfectly designed at the outset… and that any changes investigators make to a trial protocol or analytic procedures after the trial start date indicate bad science.” (Annals Editors critique, 01/03/16)	COMPare uses CONSORT as the gold standard. CONSORT item 6b requires that trial reports should declare and explain “any changes to trial outcomes after the trial commenced, with reasons” in the paper reporting the results of the trial. Changes are not forbidden, however they should be declared in the trial report.
	"The Centre for Evidence-Based Medicine Outcome Monitoring Project’s assessments appear to be based on the premise that trials are or can be perfectly designed at the outset" (Annals Editors critique, 01/03/16)	
	"Prespecification can be misused to sanctify both inappropriate endpoints, such as biomarkers, when actual health outcomes are available and methods that are demonstrably inferior." [sic] (Annals Editors critique, 01/03/16)	
Stating that outcome switching doesn’t matter if the main results of the study are unlikely to be affected.	“We reviewed materials associated with the articles and concluded that the information reported in the articles accurately represented the scientific and clinical intent detailed in the protocols... We found no inconsistencies between the audited articles and their related protocols that would justify changes in trial interpretation, corrections, or warnings to readers.” (Trial 45, Annals, 06/04/16)	CONSORT requires all outcomes to be correctly reported; it does not distinguish between circumstances when this would, or would not, affect the overall interpretation of the intervention being trialled. It is unlikely that all outcome misreporting would change the direction or size of an overall finding; however a culture of permissiveness around correct outcome reporting does permit misrepresentation more broadly.
	"This is true [secondary outcome SVR24 not reported in publication] but justifiable. In NEJM letters to editor about this paper (in press), the authors reported that they have analyzed the SVR24 data and results are equivalent to SVR12 (i.e. there were no relapses between week 12 and week 24 post treatment) (NEJM first comments on trial 22 (3))	
Statement describing journal practices that contradict CONSORT guidance.	"We support transparent and accurate reporting and, in particular, require the reporting of the most clinically relevant outcomes used to justify claims of efficacy or harm." (Trial 45, Annals, 06/04/16)	CONSORT item 6b requires that trial reports should declare and explain “any changes to trial outcomes after the trial commenced, with reasons” in the paper reporting the results of the trial.
	“We view each piece individually and add the data as appropriate based on the judgment of the peer reviewers, the statistical reviewers, and the editors.” (NEJM emails 1, 17/11/15)	
Statement that failure to report prespecified secondary outcomes is not of interest.	"We will not ordinarily consider letters that simply... point out unpublished secondary outcomes." (JAMA emails, 09/12/15)	
Denial of endorsing CONSORT, despite appearing on CONSORT’s list of endorsing journals.	“The New England Journal of Medicine finds some aspects of CONSORT useful but we do not, and never have, required authors to comply with CONSORT.” (NEJM emails 1, 17/11/15)	
**Criticisms of COMPare methods and data**		
Misrepresentation of COMPare’s methods	“The initial trial registry data… serve as COMPare’s “gold standard”.” (Annals Editors critique, 01/03/16)	This is untrue. As explained in our publicly accessible operations manual, COMPare used the registry entry where there was no pre-commencement protocol publicly available, as CONSORT 6b requires that changes after commencement are noted in the trial report. Notably, no Annals trial had a publicly accessible pre-commencement protocol.
	COMPare's method is a “simple check for an exact word match between outcomes entered in a registry and those reported in a manuscript, but that oversimplifies a highly nuanced process.” (Annals to BMJ).	This is untrue. COMPare did not seek literal word matches: each prespecified outcome was manually checked and re-checked, as per previous research on outcome misreporting, using CONSORT as gold standard.
Invalid criticism of COMPare data point.	“Our review indicates problems with COMPare’s methods. For one trial COMPare apparently considered the protocol published well after data collection ended.” (Annals Editors critique, 01/03/16)	This is untrue. COMPare used the registry entry for this trial, because the protocol was published after the trial started, and after data collection ended, as is clear on the data sheet.

	"[The criticism by COMPare that] AEs leading to discontinuation [were] not correctly reported... is false. Protocol indicates safety and tolerability as second of 2 primary objectives, and registration lists incidence of AEs leading to discontinuation as 1 of 2 primary outcome measures. First line of Table 3 and first sentence of Safety section (p. 2604) reports that 1 of 624 patient treated with sofosbuvir--velpatasvir discontinued due to AE." (NEJM first comments on trial 22 (1))	This is invalid. The outcome in question was prespecified as a primary outcome, but incorrectly reported by NEJM as a secondary outcome. COMPare therefore coded it as reported, but incorrectly reported. This is clearly denoted in the COMPare assessment sheet for this trial, and the COMPare letter reads "There were 2 pre-specified primary outcomes, of which one is reported in the paper; while one is incorrectly reported as a secondary outcome."
	"[The criticism by COMPare that] Secondary outcome SVR [was] not reported in publication... is false. This is reported in Table 2. The COMPARE reviewers may not appreciate that SVR4 (sustained virologic response week 4) is equivalent to HCV RNA <15 IU/ml at week 4, which is reported in Table 2. HCV RNA <15 IU/ml is the lower limit of detection of the assay, as indicated in the Table footnote." (NEJM first comments on trial 22 (2))	This is invalid. COMPare correctly coded this outcome as missing. Table 2 does report HCV RNA < 15 IU/ml at "week 4" but this was week 4 during treatment (which was 12 weeks long); SVR4 is Sustained Virologic Response at week 4 post-treatment. Hence, we correctly concluded that HCV RNA <15 IU/ml at week 4 post-treatment (SVR4) was not reported in the publication. It seems NEJM editors did not realise that SVR4 is 4 weeks post--treatment, rather than the 4th week of treatment, hence their misunderstanding and misreporting of this outcome in NEJM, and their error in their review of the letter from COMPare.
	"[The criticism by COMPare that] proportion with HCV RNA <LLOQ on treatment [was] not reported in publication… is false. The COMPARE reviewer may not appreciate that "HCV RNA <LLOQ" is equivalent to "HCV RNA <15 IU/ml." Table 2 reports HCV RNA <15 IU/ml during treatment." (NEJM first comments on trial 22 (4))	This is invalid. The time-point for this outcome was given in the registry entry as "up to 8 weeks", and results were reported in NEJM only for 2 and 4 weeks. We therefore concluded that the prespecified outcome was not reported. the fact that this discrepancy relates only to the timepoint is made explicit in the letter submitted by COMPare to NEJM, which states that the outcome "is not reported at the pre-specified timepoint, but is reported at two novel time-points". Because of variation in clinical presentation over time, and the attendant risk of selective reporting, under CONSORT each separate timepoint at which an outcome is measured is regarded as a separate outcome.
	"[The criticism by COMPare that] HCV RNA change from baseline [was] not reported in publication… is false. The change in HCV RNA from baseline is conveyed by reporting the mean HCV RNA at baseline (Table 1) and the rates of HCV RNA <15 IU/ml (Table 2). Table S4 reports the HCV RNA levels for the 2 patients who virologic failure." (NEJM first comments on trial 22 (5))	This is invalid, and represents a concerning approach to reporting pre-specified outcomes. NEJM suggests readers should subtract one number from another to calculate the results for a prespecified outcome themselves. In addition "HCV RNA change from baseline" cannot be calculated from the numbers reported. Mean baseline HCV RNA is reported. Mean follow-up HCV RNA is not reported. Table 2 reports only the proportion of patients with HCV RNA <LLOQ (undetectably low).
	"[The criticism by COMPare that] proportion with virologic failure [was] not reported in publication… is false. This is reported in Table 2 which reports virologic failure during treatment (0 patients) and virologic failure after treatment (1 patient)." (NEJM first comments on trial 22 (6))	This is invalid. COMPare coded this outcome as "correctly reported". This is clear on the assessment sheet.
Claim that COMPare coding incorrect on specific outcomes	NEJM gave journalists a detailed review of COMPare's assessment of one trial, which NEJM stated had identified six errors in COMPare's assessment. This was reviewed, and NEJM were wrong on all six counts, full details are given in the table above and in the correspondence appendix (NEJM first comments on trial 22). Another NEJM review of a COMPare letter was also factually wrong on all three issues it raised (NEJM second comments on trial 22 (2)).	The editors were wrong on all nine issues raised. The document they sent exemplified misunderstandings around the importance of reporting all prespecified timepoints for each prespecified outcomes.
Warning readers against COMPare's assessments	Until the COMPare Project's methodology is modified to provide a more accurate, complete and nuanced evaluation of published trial reports, we caution readers and the research community against considering COMPare's assessments as an accurate reflection of the quality of the conduct or reporting of clinical trials. (Annals Editors critique, 01/03/16), (Trial 25, Annals, 14/12/15), (Trial 44, Annals, 15/12/16), (Trial 45, Annals, 15/12/15), (Trial 68, Annals, 30/12/15),	Following this comment no trialists engaged with any of our evidence of failure to correctly report prespecified outcomes. We regarded this as a breach of ICMJE guidance as per Table 3 and discussion.
**Timing of prespecification**		
Stating or implying that prespecification after trial commencement is [illegible]	"We disagree with COMPare's contention that registry data are superior to protocol information because of the timing of the former ... " (Trial 45, Annals, 06/04/16)	Prespecification of outcomes should take place before trial commencement. CONSORT item 6b requires that trial reports should declare and explain "any changes to trial outcomes after the trial commenced, with reasons" in the paper reporting the results of the trial.

acceptable.	COMPare "did not consider the protocol published 2 years before MacPherson and associate’s primary trial was published... The protocol for MacPherson’s trial was more specific in describing the timing of the primary outcome (assessment of neck pain at 12 months) than the registry (assessment of neck pain at 3, 6 and 12 months), yet COMPare deemed the authors’ presentation of the 12 month assessment as primary within the trial publication to be “incorrect”... Furthermore, the protocol for that trial clearly listed the secondary outcomes that COMPare deemed as being not prespecified." (Annals Editors critique, 01/03/16)	This protocol was published two years before the trial's results, but one year after the trial's commencement. CONSORT requires that discrepancies are reported against outcomes prespecified before a trial's commencement.
	“The letters you submitted are for the most part not consistent with the approach outlined in your website, “comparing the clinical trials registry and trial protocol with the trial report.” Most of the letters have noted discrepancies between the trial registry and trial report, but it appears that you have not always checked for discrepancies with the trial protocols, which have been included as a supplement with each trial published in JAMA since mid-2014.” (JAMA emails, 9/12/15)	Protocols were used, unless there was no pre-commencement protocol available, in which case the registry entry was used. This is because CONSORT requires that discrepancies are reported against outcomes prespecified before a trial's commencement.
**Registries**		
Dismissal of registry data	“The initial trial registry data… often include outdated, vague or erroneous entries.” (Annals Editors critique, 01/03/16)	The statement that registry data is "outdated" may reflect a broader misunderstanding about the need for outcomes to be prespecified pre-commencement, as discussed with further examples of statements from Annals editors in the appropriate section of this table. The suggestion that registry entries are "vague or erroneous" is notable: trialists are legally required to register their trials and prespecified outcomes are a compulsory field for all registries under WHO guidance. If "erroneous" is intended to mean "discrepant with a contemporaneous protocol" then this raises concerns about the phenomenon identified by COMPare of multiple sets of discrepant but contemporaneous prespecified outcomes for some trials, as discussed elsewhere in this paper and in the paper on trialists' responses.
	“We check the registries, but as both authors’ responses attest, registry information can be incomplete or lack sufficient detail, and we rely more heavily on the protocol that guided the actual conduct of the trial.” (Trial 45, Annals, 06/04/16).	Trialists are legally required to correctly register their trials, and ICMJE member journals commit to ensuring trials are appropriately registered. Where outcomes are so poorly prespecified that correct outcome cannot be assessed, we suggest this is noted in the paper reporting the trial's results, as it presents a similar risk of bias to misreporting of clearly prespecified outcomes.
	“We carefully check for discrepancies between the protocol and the manuscript… In our experience, the trial registration may not always accurately reflect the protocol, especially if clearly documented, justified, and approved revisions to the protocol have occurred.” (JAMA emails, 9/12/15)	Different contemporaneous sources of prespecified outcomes should be consistent; if they are not this is concerning, and throws doubt on the whole notion of prespecification. For one trial [Trial 57, Annals, 03/05/16] we found three different sets of prespecified outcomes in two registries (EUCTR and clinicaltrials.gov) and one protocol from the same time period. Changes to prespecified outcomes after trial commencement are acceptable, but should be declared in the trial report as per CONSORT 6b. Both these comments from editors suggest that the rejection of registry outcomes is partly because a post-commencement source of outcomes was regarded as superior. Note that COMPare used registry entries for prespecified outcomes only where they could not be sourced from pre-commencement protocols.
	“Registries include only extracted information, do not routinely monitor whether the data in the registry match the protocol, and may not be updated when the protocol changes. We therefore rely primarily on the protocol” (Annals Editors critique, 01/03/16)	
	"We will not ordinarily consider letters that simply note discrepancies with the trial registration." (JAMA emails, 09/12/15)	Where the registry entry is the only accessible source of prespecified outcomes, discrepancies should be declared as per CONSORT 6b. Even if there is a contemporaneous protocol that is not publicly accessible, the prespecified outcomes in this protocol should match its registry entry; if not then there are two sets of discrepant prespecified outcomes, which requires declaration and discussion.
Stating that discrepancies between outcomes prespecified in a registry entry, and those reported in the paper, are the fault of the registry.	"Inaccuracies in the trial registration documents are more of an issue for the individuals overseeing the trial registries." (JAMA emails, 9/12/15)	It is the responsibility of the journal and trialist to ensure a trial is correctly reported, with discrepancies against outcomes prespecified prior to commencement declared as per CONSORT 6b. If there are discrepancies between the outcomes prespecified and the outcomes reported in the paper, then the paper is discrepant, not the source of prespecified outcomes.
**Rhetoric**		
Stating that space constraints prevent all [illegible]	“Space constraints for articles published in the Journal do not allow for all secondary and other outcomes to be reported” (NEJM emails 1, 21/11/15)	The claim that space constraints prevent all prespecified outcomes being reported conflicts with the finding of

pre-specified outcomes being reported.	JAMA: “authors are not always required to report all secondary outcomes and all pre-specified exploratory or other outcomes in a single publication, as it is not always feasible given the length restrictions to include all outcomes in the primary report.” (JAMA emails, 9/12/15)	[illegible] COMPare, and prior research on outcome misreporting, that non-prespecified additional outcomes were routinely added (mean 5.4 outcomes added per trial in COMPare, range 2.9-8.3 by journal).
General statement about supporting goals of COMPare.	Though we share COMPare’s overarching goals to assure the validity and reporting quality of biomedical studies, we do not agree with their approach. (Trial 44, Annals,15/12/16)<br><br>While the goal of the COMPare project (http://www.compare-trials.org) is noble, my colleagues and I have outlined concerns with COMPare’s approach (1), (Trial 45, Annals, 06/04/16)<br><br>“We share COMPare’s overarching goals to assure the validity and reporting quality of biomedical studies, but we differ on how to best achieve those aims.” (Annals Editors critique, 01/03/16)	All such statements were accompanied by caveats, generally references to methodological criticisms that were invalid, statements that explicitly or implicitly undermined the journals' commitment to CONSORT, or incorrect statements about specific data points.
Reasons for rejecting COMPare letters.	"In addition, some of the information in your letters is vague, containing only numbers and not specific outcomes, making it difficult to understand the specific issues or reply to them. Moreover, the last 2 paragraphs of the letters you have submitted, concerning CONSORT and the COMPare project, are identical." (JAMA emails, 09/12/15)	All correction letters linked to the COMPare online repository where all underlying data was shared in full. This letter was received halfway through the study period. To address the reasons given for letter rejection, all subsequent letters had no repetition and extensive detail within the text on specific misreported outcomes. However none of these subsequent letters were published and we received no further replies, as per Table 3.
**Statements about journal processes**		
Statement that authors are required to declare changes to outcomes.	“When the review process generates requests for authors to report outcomes not specified in the protocol or the authors choose themselves to present such outcomes, we ask authors to indicate these as post hoc or exploratory analyses.” (Annals Editors critique, 12/02/16)<br><br>“To be consistent with CONSORT recommendations, we ask authors to describe, either in the manuscript or in an appendix, any major differences between the trial registry and protocol, including changes to trial endpoints or procedures.” (Annals Editors critique, 01/03/16)	We cannot verify whether Annals ask authors to do this; however we can confirm that trials reported in Annals are routinely non-compliant with CONSORT, a finding which is consistent with previous research. COMPare found that in Annals trials: 6% of novel outcomes added to trial reports were correctly indicated as novel; a mean of 6.4 novel undeclared outcomes were added per trial; 44% of primary outcomes were correctly reported; and 31% of secondary outcomes correctly reported.
Statement that journal has a process to ensure correct outcome reporting	"We carefully check for discrepancies between the protocol and the manuscript", (JAMA emails, 09/12/15)<br><br>“We agree that it is important for researchers to pre-specify primary and secondary outcomes before conducting a trial and to report outcomes accurately in their publications. In fact, we carefully monitor this during editorial review.”(JAMA emails, 9/12/15)	We cannot verify JAMA's internal processes; however we can confirm that trials reported in JAMA are routinely non-compliant with CONSORT, a finding which is consistent with previous research. COMPare found that in JAMA trials: 39% of novel outcomes added to trial reports were correctly indicated as novel; a mean of 4.1 novel undeclared outcomes were added per trial; only 82% of primary outcomes were correctly reported; and 70% of secondary outcomes correctly reported.
**Placing responsibility on others (e.g., trialists or reader)**		
Passing responsibility to trialists, rather than journals or editors.	The Lancet published 15/20 letters, mostly with accompanying responses from trialists: the majority of author responses expressed further misunderstandings about what constitutes correct outcome reporting, as reported in the accompanying paper on trialists' responses. The Lancet made no comment themselves [all correspondence]. We asked the journal to clarify their position in our follow-up correspondence: “Since The Lancet have a longstanding positive commitment to improving reporting standards, lead the REWARD campaign on research integrity, and endorse CONSORT, we would welcome their perspective on why undeclared outcome switching in PETIT2 (and others) was apparently not addressed prior to publication; whether they now view outcome switching as acceptable; or whether they disagree that it has happened here.” We received no reply and our letter was not published. (Trial 9, Lancet, 05/02/16).	Where a journal is listed as endorsing the CONSORT guidelines on trial reporting it is reasonable to expect that they will take responsibility for ensuring trials are reported consistently with these guidelines.
Placing responsibility on trial registry staff	"Inaccuracies in the trial registration documents are more of an issue for the individuals overseeing the trial registries." (JAMA emails, 9/12/15)	As above, if there are discrepancies between the outcomes prespecified and the outcomes reported in the paper, then the paper is discrepant, not the source of prespecified outcomes.

Stating that readers can see for themselves if outcomes reported are discrepant with those pre-specified.	NEJM “Any interested reader can compare the published article, the trial registration and the protocol (which was published with the article) with the reported results to view discrepancies” (NEJM emails 1, 21/11/15)	COMPare found that accessing documents and assessing trials for correct outcome reporting took between one and seven hours per trial.
**Positive responses to COMPare**		
Issuing a correction.	The BMJ issued a 149 word correction on the REEACT trial after receiving COMPare's correction letter (REEACT correction, BMJ, 12/01/16).	Out of 756 breaches of CONSORT identified by COMPare across 58 trials these were the only two corrections issued.
	After COMPare attempted to access a protocol for a trial published in Annals from the lead author, we received an email from the sponsor telling us access was only possible if we signed a confidentiality agreement. This conflicted with the written transparency commitment made in the Annals publication to share the protocol on request. Annals issued a correction. "The “Reproducible Research” statement accompanying the article was incomplete. It indicated that the protocol was available by contacting the authors but should also have stated that signature of a confidentiality agreement was necessary to obtain the protocol." (Trial 45, Annals, 12/04/16)	
**Notes**		
References throughout are to COMPare-trials.org/data, containing the full correspondence on all trials, organised by Trial ID and date, or Journal Name for general correspondence.		
