# Supplementary material for: COMPare: a prospective cohort study correcting and monitoring 58 misreported trials in real time
Source: Trials. 2019 Feb 14;20:118. doi: 10.1186/s13063-019-3173-2 (PMC6375128; doi:10.1186/s13063-019-3173-2)
Supplement: Supplementary file 1 — a General template letter. b Template letter to the New England Journal of Medicine (NEJM). c Template letter to The Lancet. (ZIP 20 kb) [file 13063_2019_3173_MOESM1_ESM.zip › Letter Template - COMPare studyR1.docx]

Dear Editor,

Your recent publication [Author] *et al* [1] reports outcomes that are different to those initially registered [2].

There was [ ] pre-specified primary outcome, of which [ ] is reported in the paper; while [ ] is not reported anywhere in the publication. In addition, the paper reports [ ] “primary outcomes” that were not pre-specified, without flagging them as such. There were also [ ] pre-specified secondary outcome, of which [ ] were reported in the paper; while [ ] are not reported anywhere in the publication. In addition, the paper reports [ ] new secondary outcomes, which were not pre-specified, without declaring them as such.

[*Insert Name of Journal* ] has endorsed the CONSORT guidelines on best practice in trial reporting [3]. In order to reduce the risk of selective outcome reporting, CONSORT includes a commitment that all pre-specified primary and secondary outcomes should be reported; and that, where new outcomes are reported, it should be made clear that these were added at a later date, with an explanation of when and for what reason. We have attached an annotated version of the results table (Figure 1), and a corrected results table (Table 1) reporting the outcomes that were pre-specified for the trial where those are available, or with the word “missing” where those pre-specified outcomes are not reported anywhere.

This letter has been sent as part of the COMPare project [4]. We aim to review all trials published from now in a sample of top journals, including *[name of Journal]*. Where outcomes have been incorrectly reported we are writing letters to correct the record, and to audit the extent of this problem, in the hope that this will reduce its prevalence. We are maintaining a website at [COMPare-Trials.org](http://compare-trials.org/) where we will be posting the submission date and publication date of this letter, alongside a summary of the data on each trial and journal. We hope that you will publish this letter so that those using the results of this trial to inform decision-making are aware of the discrepancies.

Yours faithfully,

[*First Author*], [*Second Author*], [Senior academic] on behalf of the COMPare project team.

[1] Trial report

[2] Trial registry entry

[3] Moher D et al, CONSORT 2010 Explanation and Elaboration: updated

guidelines for reporting parallel group randomised trials, BMJ 2010; 340:c869.

[4] COMPare project website [www.COMPare-Trials.org](http://www.compare-trials.org)

*Figure 1 : Annotated results table - ‘P1’ signifies a pre-specified primary outcome, ‘P2’ specifies a pre-specified secondary outcome, and ‘U’ specifies a non-pre-specified outcome.*
