# Supplementary material for: COMPare: a prospective cohort study correcting and monitoring 58 misreported trials in real time
Source: Trials. 2019 Feb 14;20:118. doi: 10.1186/s13063-019-3173-2 (PMC6375128; doi:10.1186/s13063-019-3173-2)
Supplement: Supplementary file 1 — a General template letter. b Template letter to the New England Journal of Medicine (NEJM). c Template letter to The Lancet. (ZIP 20 kb) [file 13063_2019_3173_MOESM1_ESM.zip › Lancet Letter Template - COMPare studyR1.docx]

Dear Editor,

Your recent publication [Author] *et al* [1] reports outcomes that are different to those initially registered [2].

There were A pre-specified primary outcomes, of which B were reported in the paper; while C are not reported anywhere in the publication. In addition, the paper reports D new primary outcomes that were not pre-specified, without flagging them as such. There were E pre-specified secondary outcomes, of which F were reported in the paper; while G are not reported anywhere in the publication. In addition, the paper reports H new secondary outcomes that were not pre-specified, without flagging them as such.

The Lancet has endorsed the CONSORT guidelines on best practice in trial reporting, which aim to reduce the risk of selective outcome reporting [3]. Our team is currently tracking all trials in five top journals to monitor and signpost incorrect outcome reporting, as part of the COMPare project [4]. We are maintaining a website at COMPare­-Trials.org where we will be posting the submission date and publication date of this letter, alongside a summary of the data on each trial and journal.

Attached is an annotated version of the results tables (Figure 1), showing which of the outcomes reported in this trial were pre-specified, and which were not. [*OR:* ...a corrected results table (Figure 1), giving the results for the pre-specified outcomes, or the words “data not available”]. We hope you will publish this letter to ensure those using the results of this trial are aware of the discrepancies.

Yours faithfully,

[Assessor 1], [Assessor 2], [Senior academic] (Up to 5 authors in total) on behalf of the COMPare project team.

[1] Trial report

[2] Trial registry entry

[3] Moher D et al, CONSORT 2010 Explanation and Elaboration: updated

guidelines for reporting parallel group randomised trials, BMJ 2010; 340:c869.

[4] COMPare project website [www.COMPare-Trials.org](http://www.compare-trials.org)
