# Supplementary material for: COMPare: a prospective cohort study correcting and monitoring 58 misreported trials in real time
Source: Trials. 2019 Feb 14;20:118. doi: 10.1186/s13063-019-3173-2 (PMC6375128; doi:10.1186/s13063-019-3173-2)
Supplement: Supplementary file 1 — a General template letter. b Template letter to the New England Journal of Medicine (NEJM). c Template letter to The Lancet. (ZIP 20 kb) [file 13063_2019_3173_MOESM1_ESM.zip › NEJM Letter Template - COMPare studyR1.docx]

Dear Editor,

Your recent publication [Author] *et al* [1] reports outcomes that are different to those initially registered [2].

There were A pre-specified primary outcomes, of which B were reported in the paper; while C are not reported anywhere in the publication. In addition, the paper reports D new primary outcomes that were not pre-specified, without declaring them as such. There were E pre-specified secondary outcomes, of which F were reported in the paper; while G are not reported anywhere in the publication. In addition, the paper reports H new primary outcomes that were not pre-specified, without declaring them as such.

Our team is currently tracking all trials in five top journals to monitor and signpost incorrect outcome reporting, as part of the COMPare project [3].

Attached is an annotated version of the results tables (Figure 1), showing which of the outcomes reported in this trial were pre-specified, and which were not. [*OR:* ...a corrected results table (Figure 1), giving the results for the pre-specified outcomes, or the words “data not available”]. We hope you will publish this letter to ensure those using the results of this trial are aware of the discrepancies.

Yours faithfully,

[Assessor 1], [Assessor 2] and Ben Goldacre on behalf of the COMPare project team.

[1] Trial report

[2] Trial registry entry

[3] COMPare project website [www.COMPare-Trials.org](http://www.compare-trials.org)
